# Supplementary material for: Increased Levels of cAMP by the Calcium-Dependent Activation of Soluble Adenylyl Cyclase in Parkin-Mutant Fibroblasts
Source: Cells. 2019 Mar 15;8(3):250. doi: 10.3390/cells8030250 (PMC6468892; doi:10.3390/cells8030250)
Supplement: Supplementary file 1 [file cells-08-00250-s001.pdf]

Supplemental Table S1: Primer Sequences

Gene	Forward Primer	Reverse Primer
**PDE4A**	5′GTGGCTCCGGATGAGTTCTC 3′	5′ GGGCTGCTGTGGCTTACAG 3′
**ADCY3**	5′ GGCAGCTAAATACCACCAGC 3′	5′ ATGAGGATGGAGCAGACGG 3′
**ADCY6**	5′ AGCTCTGTCTTCCTGCACAT 3′	5′ ACGCCAAGCAGTAGGTCATA 3′
**ADCY10**	5′ GCAGAAGAGATAGGCCCAGC 3′	5′ AGAGCAGCCCTTGTCAAACA 3′
**GAPDH**	5′GAAGGTGAAGGTCGGAGT3′	5′CATGGGTGGAATCATATTGGAA3′
